# Assessment of Novel Water Applied Prebiotic to Evaluate Gut Barrier Failure and Performance in Two Commercial Trials in Brazil. A Pilot Study With an Economic Perspective

**DOI:** 10.3389/fvets.2021.652730

**Published:** 2021-06-08

**Authors:** Igor Praxedes-Campagnoni, Bruno Vecchi, Emanuel Gumina, Xochitl Hernandez-Velasco, Jeffrey W. Hall, Sherry Layton

**Affiliations:** ^1^Vetanco SA, Villa Martelli, Argentina; ^2^BV Science, Lenexa, KS, United States; ^3^Departamento de Medicina y Zootecnia de Aves, Facultad de Medicina Veterinaria y Zootecnia, Universidad Nacional Autonoma de Mexico, Ciudad de Mexico, Mexico; ^4^Vetanco USA, Saint Paul, MN, United States

**Keywords:** prebiotic, broiler chicken, immunomodulator, intestinal integrity, performance

## Abstract

The present study evaluated the effect of administration of a water applied prebiotic on gut barrier failure (Experiment 1) and performance in broiler chickens under commercial conditions (Experiment 2). Experiment 1, one thousand four hundred and forty day-of-hatch Ross broiler chickens were assigned to one of two experimental groups (*n* = 30 replicate pens/treatment; *n* = 24 chicks/pen). Birds in the treated group received the prebiotic orally in the drinking water (0.2ml/bird) on days 3 and 17 of age. The second group served as the untreated control group. On d 18, intestinal samples were analyzed by qRT-PCR to determine the expression of *MUC2, IL-8, TGF-*β*4*, and *ZO-1*. On d 17, d 28, and d 35 blood samples were collected to determine circulating endotoxin levels. On d 28, mucosal intestinal scrapping was collected to measure relative total sIgA levels. At d 42, liver samples were collected to evaluate liver bacterial translocation. In Experiment 2, the prebiotic was evaluated in two commercial trials. Chickens were raised under normal production conditions and fed a 3-phase commercial basal diet with enramycin (7 g/ton). In Trial 1, 8,974,237 broiler chickens were treated with the prebiotic. The prebiotic was administered in the drinking water (0.2 mL/bird) following the manufacture label instructions at day three and seventeen of life. Production parameters were compared to historical information from the company over the same broiler operation and production cycles. For trial 2, 921,411 broiler chickens were treated with the prebiotic as in Trial 1. In Experiment 1, treated chickens showed a significant (*P* < 0.05) increase in mRNA expression of *MUC2, TGF-*β*4, IL-8, ZO-1*, and sIgA, but a significant reduction of serum endotoxin levels and incidence of liver lactose positive bacterial translocation when compared to non-treated chickens. In both trials of Experiment 2, a significant reduction in total mortality was observed in the treated chickens when compared with the historical farm data. Economic analysis utilizing the total percent of mortality revealed a $1: $2.50 USD and $1: $4.17 USD return for Trial 1 and Trial 2, respectively. The results suggest that the prebiotic positively influences gastrointestinal integrity and performance.

## Introduction

The primary functions of the gastrointestinal tract are limited to digestion and absorption of nutrients and water is a statement used frequently. However, nothing can be further from the truth. Today, the enteric nervous system, consisting of over one hundred million neurons, is considered the “second brain” of animals (1). The gut-associated lymphoid tissue (GALT) contains over 70% of the total immune cells of the body and about 80% of all plasma cells, which are primarily immunoglobulin A (IgA)-producing cells. (2). Furthermore, the gastrointestinal tract microbiota complements the biology of metazoans, and is considered another “organ” within the intestine with the number of microbial cells out numbering total number of somatic cells and genes in a proportion of 10:1 (3–5). The astonishing neuroendocrine network between the central nervous system, the enteric nervous system, the intestinal microbiota and the GALT, have a substantial impact on the fragile intestinal epithelial barrier. This barrier, consisting of a single layer or enterocytes, with its intercellular tight junctions (TJ), controls the balance between tolerance and immunity to non-self-antigens (6–8).

Advances in molecular biology, analytics, and data science have helped the scientific community gain a deeper understanding of the interactions between nutrition, the microbiota, and the immune system (6). In parallel, the global trend to reduce antibiotic growth promoters (AGP) in the poultry industry has gathered momentum (9). Currently, the world's population is more conscious than ever about diseases associated with animal production, especially the zoonotic diseases that affect public health. There is a continual emphasis on antibiotic resistance associated with these potentially zoonotic microorganisms, many of which influence human medicine. These consumer concerns have forced governments to create new regulations and address replacing antibiotics with alternatives that provide similar benefits but do not pose the same public health risks (10). These trends supported the development of a novel water applied prebiotic (Gamaxine®). The concept of prebiotics developed in response to the notion that nondigestible food ingredients (e.g., nondigestible oligosaccharides) are selectively fermented by one or more bacteria known to have positive effects on gut physiology (11). Bacteria fed by a preferential food substrate have a proliferative advantage over other bacteria. Some prebiotics have shown to selectively stimulate the growth of endogenous lactic acid bacteria (LAB) in the gut to improve the health of the host (12). In this notion, prebiotics may have more benefits compared with live cultures that provide beneficial health properties to the host (probiotics), in that prebiotics stimulate commensal bacteria which have adapted to the environment of the gastrointestinal tract (13). Prebiotics have been reported to enhance host defense and reduce mortality of birds caused by the invasion of gut pathogens (14–17). The mechanism by which prebiotics exert this feature remains less elucidated, but it is likely that the capacity of prebiotics to increase the number of LAB in the gut may aid the competitive exclusion of pathogens from the gastrointestinal tract of birds (12). The increased production of short chain fatty acids with the administration of prebiotics resulting in increased intestinal acidity may also contribute to the suppression of pathogens in the gut of chicken (18). Prebiotics have also been reported to enhance the immune response of chicken (19). The most common prebiotics used in poultry are oligosaccharides, including inulin, fructooligosaccharides (FOS), mannan-oligosaccharides (MOS), galactooligosaccharides (GOS), soya-oligosaccharides (SOS), xylo-oligosaccharides (XOS), pyrodextrins, isomaltooligosaccharides (IMO), and lactulose (20, 21). Studies have confirmed that prebiotic selectively modify the colonic microflora and can potentially influence gut metabolism, digestion and absorption of nutrients (22–24). Further, the use of intact, or components, of the Gram-positive bacteria, *Bacillus subtilis*, has shown to have positive effects on intestinal barrier integrity, reduce inflammatory signals, promote intercellular junctions, and improve animal performance (25–28). Several studies have confirmed that the use of prebiotics in poultry diets improve performance and reduce food borne pathogens (29–32). However, no studies have evaluated the delivery of a *Bacillus subtilis-*based prebiotic in the drinking water and its economic impact under field trial conditions. Hence, the objectives of the present study were to evaluate the effect of this novel water applied prebiotic on gut barrier failure and performance in broiler chickens under large scale commercial conditions.

## Materials and Methods

### Prebiotic

Gamaxine® (BV Science, Inc. Lenexa, KS 66219) is a water applied prebiotic made up of *Bacillus subtilis* cells and fermentation products, yeast cell wall components, and mannan-oligosaccharides (MOS). After fermentation, *Bacillus subtilis* is chemically inactivated. The proteinaceous structural components of the bacteria and the metabolites secreted during the fermentation are combined with yeast cell wall components, and mannan-oligosaccharides (MOS) in a proprietary formulation for delivery *via* drinking water.

### Experiment 1. Evaluation of the Prebiotic on Body Weight Gain, Feed Conversion Ratio, Mucosal Cytokines Response, Serum Endotoxin Levels, Liver Bacterial Translocation, and Total Mucosal sIgA in Broiler Chickens

This experiment was conducted at the experimental farm of Bioinnovo in Buenos Aires, Argentina. The broiler barn is an open-sided 600 square meter facility with a concrete floor, 60 (2 × 2m^2^) pens with individual waterers and feeders. Heat is provided *via* air heaters and the facility has six cooling/exhaust fans. In this study, each pen contained 15 cm of wood shavings [50% reused litter (from negative control pens only) and 50% new wood shavings] to simulate commercial conditions. One thousand four hundred and forty day-of-hatch Ross male broiler chickens were individually tagged, weighed, and randomly assigned to one of two experimental groups (*n* = 30 replicate pens/treatment; *n* = 24 chicks/pen) in this experiment. Birds in the treated group received the prebiotic orally in the drinking water (0.2 ml/bird) on days 3 and 17 of age. The second group served as the untreated control group. On d 18, 24 h after the second dose of prebiotic, individual intestinal illeal samples were collected (*n* = 10/group), total RNA isolated and analyzed by qRT-PCR to determine the expression level of mucin 2 (*MUC2*), interleukin 8 (*IL-8*), transforming growth factor β4 (*TGF-*β*4*) and zonula occludens-1 (*ZO-1*). On d 17, d 28, and d 35 blood samples were collected (*n* = 25/group) to determine circulating endotoxin levels (lipopolysaccharides). On d 28, mucosal intestinal scrapping was collected to measure total sIgA in the gastrointestinal tract (*n* = 25/group). Upon the experiment's conclusion, d 42, liver samples (*n* = 15) were collected to evaluate liver bacterial translocation. At each sampling time, one chicken was randomly selected from the 30 replicate/treatment.

#### Mucosal Cytokine mRNA Expression Measured by qRT-PCR

Briefly, a 10 cm section of the ileum starting ~2 cm above the ileo-ceca junction was collected and rinsed with ice-cold phosphate-buffered saline (pH 7.4) and cut open to scrape mucosa using RNase-free glass slides into 2-mL RNase and DNase-free sterile tubes. The mucosal scrapings were placed on ice and then transferred to −20°C for storage until total RNA isolation. Total RNA was isolated using Qiagen RNeasy (Qiagen 74104), Qiagen Qiashredder columns (Qiagen 79654), and RNase-Free DNase Set (Qiagen 79254) according to manufacturer's suggested protocols. 50 ng of total RNA was converted into cDNA using BioScript All-in-One cDNA Synthesis Kit (Biotool B24403) according to the manufacturer's directions. The relative mRNA levels of the genes of interest were measured by quantitative polymerase chain reaction using Biotool 2x Probe qPCR Syber Green Master Mix (Biotool B21202) Applied Biosystems Step-One Plus (RT) PCR System. Table 1 shows the four primers used as biomarkers to evaluate gut barrier function according to Chen et al. (33). All primers were verified for the efficiency and linearity of amplification (33). Expression of the genes can either be upregulated in which the results will be greater than one or downregulated in which the results would be less than one as described by Livak and Schmittgen (34). For each gene of interest, an unpaired Student's *t*-test was used to compare the ΔCt for the prebiotic treated vs. untreated samples. The null hypothesis (no difference is geometric means of groups) was rejected at α ≤ 0.05. The ΔΔCt method (2^−ΔΔCT^) was used to calculate the relative gene expression between the prebiotic treated and untreated groups.

**Table 1:** List of primers used for qRT-PCR.

**Genes**	**Forward primer**	**Reverse primer**	**Fragment size (bp)**
Actin	CAACACAGTGCTGTCTGGTGGTA	ATCGTACTCCTGCTTGCTGATCC	205
MUC2	GCCTGCCCAGGAAATCAAG	CGACAAGTTTGCTGGCACAT	59
IL-8	TCCTGGTTTCAGCTGCTCTGT	CGCAGCTCATTCCCCATCT	52
TGFβ4	CGGCCGACGATGAGTGGCTC	CGGGGCCCATCTCACAGGGA	113
ZO1	CCGCAGTCGTTCACGATCT	GGAGAATGTCTGGAATGGTCTGA	63

#### Lipopolysaccharides Measurement

Bacterial endotoxin in chicken serum was quantified as an indirect measure of circulating lipopolysaccharides using the reagents supplied in the Endpoint Chromogenic Limulus Amebocyte Lysate Assay Kit QCL1000 (Lonza, Allendale, NJ, 07401) following the manufacturer's supplied instructions. In brief, blood was collected from chickens by wing vein puncture into a sterile tube, allowed to clot overnight at room temperature, centrifuged, and the serum removed and stored in a sterile, endotoxin-free microcentrifuge tube at −20°C until assayed for endotoxin. Microplates were pre-equilibrated to 37°C, while the endotoxin standard curve was prepared using the standard and reagents supplied in the kit. While leaving the microplate at 37°C, 50 μl of either the standard or serum samples were added to the appropriate wells of the microplate in duplicate. 50 μl of Limulus Amebocyte Lysate reagent water was additionally added in duplicate to wells as an absorbance control blank. Next, 50 μl of Limulus Amebocyte Lysate, previously prepared, was added to each well, and the side of the plate repeatedly tapped to facilitate mixing. The microplate was covered and incubated for 10 min at 37°C. Following incubation, 100 μl of chromogenic substrate solution, which has been prewarmed to 37°C was added to each well and incubated for an additional 6 min at 37°C. After incubation, 100 μl of stop reagent was added to each well and mixed. Each well's absorbance was then measured at 405–410 nm using a Biotek microplate reader. Mean values representing a change in absorbance were plotted against known standard concentrations generating a standard curve; sample values were calculated using linear regression analysis.

#### Liver Bacterial Translocation

To evaluate the presence of *Salmonella* spp. upon conclusion of the experiment at d 42, chickens were euthanized by cervical dislocation and liver samples (*n* = 15) were collected aseptically and placed into sterile 50 mL conical tubes containing Tetrathionate Broth (Merck) supplemented with iodine/potassium iodine for enrichment. These tubes were incubated overnight at 37**°**C, and the next day, samples from each tube were streaked aseptically onto XLD agar (Merck). These plates were then incubated at 37**°**C overnight and observed the following day for the presence of incidence of lactose negative (*Salmonella)* bacteria growth.

#### Relative Quantification of Total Mucosal sIgA (Non-specific)

For the determination of relative intestinal mucosal sIgA, the mucosal layer of a section of the ileum 2.5 cm from the cecal tonsil was cut open to scrape mucosa using RNase-free glass slides into 2-mL RNase and DNase-free sterile tubes. The mucosal scrapings were placed on ice and then transferred to −20°C for storage until total RNA isolation collected, diluted (1:1 [wt/vol]) with sterile saline, vortexed, and placed on ice. The samples were then frozen (−20°C) for storage until analysis. Just prior to use, the samples were vortexed and then centrifuged (1,000 × *g* for 15 min, 4°C), and the supernatant was used for relative sIgA determination. Mucosal samples were analyzed using components of the Chicken IgA ELISA Quantitation Kit (Bethyl Laboratories) according to the manufacturer's protocol, but the standard was omitted, in duplicate. As there were only two groups, treated (Gamaxine) and untreated, each group represents the respective positive and negative control groups. Equal volumes of mucosal sample from each respective group (Gamaxine-treated birds or non-treated birds) was pooled to create pooled positive and negative controls. Each plate also contained antigen negative wells for background signal determination. The mean absorbance obtained from these reagent blank wells were subtracted from all wells in the plate as a blank correction. The mean absorbances obtained for the pooled (Gamaxine) positive-control, pooled negative-control, and experimental samples were used to calculate sample-to-positive-control ratios using the following calculation: (sample mean – pooled negative-control mean) / (pooled positive-control mean – pooled negative-control mean). Data are presented as group average sample-to-positive-control (S/P) ratios +/− standard error of the mean (SEM).

### Experiment 2. Application of the Prebiotic to Improve Production Status in Commercial Broiler Chickens

#### Large-Scale Commercial Field in Brazil. Trial 1

For trial one, the regional complexes for one slaughterhouse from an integrated poultry producer located in Santa Catarina, Brazil was selected for the trial period of May to September 2015. In this trial, 8,974,237 broiler chickens were treated with the prebiotic over three complete production cycles (*n* = 3,046,024 chickens/production cycle), which included 485 chicken houses. This commercial company uses relevant modern genetics (Cobb, Ross, and Hubbard). However, in this trial, only farms that housed Cobb 500 males were included in both commercial trials. The prebiotic was administered in the drinking water following the manufacturer's label instructions at day three and seventeen of life. Chickens were raised under normal production conditions and fed a 3-phase commercial basal diet with enramycin (7 g/ton) which is the antibiotic growth promotor used by the company. Evaluation of production parameters was done at the end of each grow-out cycle and compared to historical information from the company over the same broiler operation and production cycles from May to September 2014 that included 488 chicken houses. Parameters evaluated included: the age of the birds at processing, final body weight (FBW), feed intake (FI), feed conversion rate (FCR), and total mortality.

#### Large-Scale Commercial Field in Brazil. Trial 2

For trial 2, the slaughterhouse with the historically worst productive results (FBW, FI, FCR and total mortality) from the same integrated poultry company located in Santa Catarina, Brazil, was selected for the trial period: May to September 2016. In this trial, 85 chicken houses, totally 921,411 broiler chickens were treated with the prebiotic. The prebiotic was administered in the drinking water following the manufacturer's label instructions at day three and seventeen of life. Chickens were raised under normal production conditions and fed a 3-phase commercial basal diet with enramycin (7 g/ton) which is the antibiotic growth promotor used by the company. Evaluation of production parameters was done at the end of each grow-out cycle and compared to historical information from the company over the same broiler operation and chicken farms during May to September 2015 that included 78 chicken houses. Parameters evaluated included: the age of the birds at processing, FBW, FI, FCR, and total mortality.

### Economic Analysis of the Large-Scale Commercial Field Trials

The economic analysis to evaluate the impact of the prebiotic under commercial conditions in Trial 1 and Trial 2 was estimated using the following formulas:

Investment = (Number of bottles used) ($12.50 USD unit cost/bottle).

Number of chickens saved = (% difference in mortality between control and treated chickens) (total number of chickens treated)/100 with the prebiotic.

kg of chickens saved = (Number of chickens saved) (average FBW of chickens treated with the prebiotic).

Profit = (kg of chicken saved) ($0.69 internal cost of production per kg).

Cost: Benefit = Profit / Investment.

### Statistical Analysis

In Experiment 1, for growth performance parameters: body weight gain (BWG), and FCR, each replicate pen was considered as an experimental unit. Performance parameters, mucosal cytokine mRNA expression by qRT-PCR, serum endotoxin levels and total sIgA, data were subjected to one-way analysis of variance (ANOVA) as a completely randomized design using the GLM procedure of SAS (35). Treatment means were partitioned using Duncan's multiple range test at α ≤ 0.05 indicating statistical significance. The incidence of lactose positive bacteria present in liver samples was compared by a chi-square test of independence at α ≤ 0.001 indicating statistical significance (36). In Experiments 2: Trials 1 and 2, the age at processing and growth performance parameters (FBW, FI, FCR, and total mortality), each chicken house of the complex evaluated, was considered as an experimental unit. All data were subjected to one-way ANOVA as a completely randomized design using the GLM procedure of SAS. Treatment means were partitioned using Duncan's multiple range test at α ≤ 0.05 indicating statistical significance.

## Results

### Experiment 1. Evaluation of a Prebiotic on Body Weight Gain, Feed Conversion Ratio, Serum Endotoxin, Gram-Negative Liver Bacterial Translocation, and Total Mucosal sIgA in Broiler Chickens

The results of the evaluation of a prebiotic on BWG, FCR, serum endotoxin values, liver bacterial translocation, and total mucosal sIgA in broiler chickens are summarized in Table 2. The birds treated with the prebiotic showed a significant increase (*P* < 0.05) in BWG (107.96 g) at d 42 when compared to the non-treated controls. Furthermore, chickens treated with the prebiotic showed a significant improvement (*P* < 0.05) in FCR (1.56 vs. 1.62) when compared with the control group. Serum endotoxin levels (0.80 ± 0.09^a^ vs. 0.21 ± 0.01^b^) and the presence of coliforms (lactose positive bacteria) in the liver (6.66 vs. 86.66%) were significantly reduced (*P* < 0.001) in treated chickens when compared with non-treated chickens. *Salmonella* spp. were not detected in either group. Additionally, total intestinal sIgA levels significantly increased (S/P ratio 2.11 vs. 0.8) in treated compared with non-treated chickens (Table 2).

**Table 2:** Evaluation of a water applied prebiotic on body weight gain (BWG), feed conversion ratio (FCR), serum endotoxin levels, liver lactose positive bacteria translocation and total mucosal sIgA in broiler chickens in experiment 1.

	**BWG, g (d 42)**	**Accumulated FCR (d 42)**	**Serum endotoxin units/mL**	**Liver translocation (Lactose positive bacteria)**	**Relative total intestinal sIgA (S/P ratio)**
Non-treated controls	3,080.71 ± 21.4^b^	1.62 ± 0.07^a^	0.80 ± 0.09^a^	13/15 (86.66 %)	0.8 ± 0.09^b^
Prebiotic	3,188.67 ± 20.7^a^	1.56 ± 0.1^b^	0.21 ± 0.01^b^	1/15 (6.66 %)^*^	2.11 ± 0.60^a^

*Data expressed as mean ± SEM. Values within columns with different superscripts differ significantly (P < 0.05). Gram-negative bacterial liver translocation data is expressed as total livers with bacterial growth on XLD agar plates/ total livers evaluated (%)*.

* *Indicates significant difference at P < 0.001*.

A significant increase in mRNA expression for *MUC2* (+2.6-fold), *TGF-*β*4* (+3.1-fold), *IL-8* (+2.3-fold), and *ZO-1* (+2.1-fold) was observed in the ileum tissues samples of the prebiotic group compared to the non-treated group (Table 3).

**Table 3:** Relative mRNA levels of genes in ileum mucosa between control and prebiotic treated broilers chickens in experiment 1.

	***MUC2***	***TGF-β4***	***IL-8***	***ZO-1***
Non-treated controls	1.01 ± 0.31^b^	1.03 ± 0.17^b^	0.99 ± 0.29^b^	1.00 ± 0.49^b^
Prebiotic	3.61 ± 0.12^a^	4.13 ± 0.71^a^	3.29 ± 0.71^a^	3.01 ± 0.11^a^

*Data expressed as mean ± SEM. Values within columns with different superscripts differ significantly (P < 0.05)*.

### Experiment 2. Application of the Prebiotic to Improve Production Status in Commercial Broiler Chickens

Table 4 shows the evaluation of the prebiotic on performance parameters in broiler chickens in two commercial trials in Brazil from Experiment 2, Trial 1 and Trial 2. In Trial 1, 9,138,072 broiler chickens were treated with the prebiotic over three complete production cycles. Chickens in those farms treated with the prebiotic had a significant reduction (*P* < 0.05) in FI (4,971.30 g ± 44.50^b^ vs. 5,132.36 g ± 12.80^a^); FCR (1.64 ± 0.0023^b^ vs. 1.67 ± 0.0021^a^) and total mortality (3.83% ± 0.07^b^ vs. 4.42% ± 0.08^a^) when compared with the non-treated historical parameters of the chickens in the same complex and same genetics (Table 4).

**Table 4:** Evaluation of a water applied prebiotic on performance parameters in broiler chickens in two commercial trials in Brazil. Experiment 2.

**Variable**	**Control**	**Prebiotic**
**Trial 1**		
Age at processing	45.98 ± 0.05	45.87 ± 0.05
Final body weight (g)	3,069.05 ± 17.29	3,066.94 ± 20.88
Feed intake (g)	5,132.36 ± 12.80^a^	4,971.30 ± 44.50^b^
FCR	1.67 ± 0.0021^a^	1.64 ± 0.0023^b^
Total mortality (%)	4.42 ± 0.08^a^	3.83 ± 0.07^b^
**Trial 2**		
Final body weight (g)	3,065.55 ± 18.9	3,049.85 ± 19.33
Feed intake (g)	5,241.15 ± 15.80	5,183.30 ± 50.60
FCR	1.71 ± 0.007	1.70 ± 0.005
Total mortality (%)	4.38 ± 0.48^a^	3.39 ± 0.07^b^

*Data expressed as mean ± SEM. Values within rows with different superscripts differ significantly (P < 0.05). Trial 1. Non-treated chicken houses n = 485; prebiotic chicken houses = 488. Trial 2. Non-treated chicken houses n = 78. Prebiotic chicken houses = 85*.

In Trial 2, chickens treated with the prebiotic had a significant reduction (*P* < 0.05) in total mortality (3.39% ± 0.07^b^ vs. 4.38% ± 0.48^a^) compared with the historical mortality of non-treated chickens in the same farms and the same genetics (Table 4).

The cost: benefit ratio of the prebiotic product in broiler chickens in two commercial trials in Brazil from Experiment 2, Trial 1 and Trial 2 are summarized in Table 5. Economic evaluation based on the total percentage of mortality revealed a $1: $2.50 USD and $1: $4.17 USD return for Trial 1 and Trial 2, respectively (Table 5).

**Table 5:** Cost: benefit ratio of a water applied prebiotic product in broiler chickens in two commercial trials in Brazil. Experiment 2, Trial 1 and Trial 2.

	**Control**	**Prebiotic**	**Difference**	**Number of chickens saved**		
**Trial 1**						
Mortality (%)	4.42 ± 0.08^a^	3.83 ± 0.07^b^	0.59%	52,948.00		
Number of bottles used	Investment	Number of chickens saved	kg of chicken saved	Chicken cost per kg	Profit	Cost: Benefit
3,590	$ 44,875.00	52,948	162,392	$ 0.69	$ 112,050.15	$ 2.50
**Trial 2**						
Mortality (%)	4.38 ± 0.48^a^	3.39 ± 0.07^b^	0.99%	9,122		
Number of bottles used	Investment	Number of chickens saved	kg of chicken saved	Chicken cost per kg	Profit	Cost: Benefit
368	$ 4,600.00	9,122	27,813	$ 0.69	$ 19,190.95	$ 4.17

*Estimated according to the numbers of broiler chickens / house treated with the prebiotic. Benefit to cost ratio = value of treatment / prebiotic product cost (expressed as cost:benefit)*.

## Discussion

The prebiotic evaluated in the present study is composed of yeast cell wall components (MOS), and fermented *Bacillus subtilis* components. Dietary supplementation of MOS in poultry diets with or without AGP has been shown to have positive benefits in terms of performance, immune modulation, and reduction of pathogens (37–41).

Analysis from the mucosal tissues lining the gastrointestinal tract of chickens in Experiment 1, demonstrated consistent upregulation of the genes involved in innate immunity (*TGF-*β*4*), inflammation (*IL-8*), and intestinal integrity (*MUC2* and *ZO-1*). MUC2 is the most abundant mucin in the intestine, and its deficiency has been reported to increase bacterial translocation and inflammation (42). TGF-β is an anti-inflammatory immune response factor and *Bacillus subtilis* strains have been shown to upregulate the expression of this cytokine (43). IL-8 is an interleukin associated with the activation of the mucosal innate immune response (44). In addition, chickens that received the prebiotic showed an increase in mucosal levels of sIgA, an essential immune defense molecule specific to the mucosa, considered an important biomarker in gut integrity (45, 46). These results suggest that the molecules present in the prebiotic helped to balance pro-inflammatory and anti-inflammatory responses, hence maintaining gut integrity. Furthermore, chickens that received the prebiotic showed an increase of *ZO-1* when compared with non-treated chickens. By increasing the expression of *ZO-1*, the prebiotic may enhance an animal's ability to withstand this increased luminal pressure, preventing microbial translocation to the circulatory system as was evidenced by a significant reduction in liver lactose positive bacteria translocation and serum endotoxin levels. The liver receives ~70% of its blood supply from the intestine through the portal circulation and is the interface between the intestinal microenvironment and the rest of the body (47, 48). Hence, the liver provides an excellent target to investigate TJ and mucosal integrity and translocation of bacteria by simple microbial plate analysis (49). Hence, through a complex series of physical, physiological, chemical, and biological mechanisms, these systems are interconnected and dynamic; continuously changing based on the situation at any given period.

The results obtained from Experiment 1 showed that the administration of the prebiotic increases total weight gain and improves feed conversion. These results were further confirmed in two commercial trials over multiple production cycles covering a 5-month period (Experiment 2). This approach provided a robust estimation of animal performance results validated at a commercial scale. In Trial 1, the prebiotic significantly reduced FI, FCR, and total mortality compared with the historical data from the company, evaluating the same genetic line. When the prebiotic was tested in those farms with the worst historical record of performance (Trial 2), a significant reduction in total mortality was observed compared with the same historical records of the complex evaluated the previous year. The economic analysis revealed positive outcomes to the poultry company, suggesting that the use of the prebiotic has economic benefits, especially in those farms that, for some reason, have historically bad performance records.

Many companies still use AGP in their commercial operations. The decision to use specific alternative feed additives such as prebiotics is governed by the costs of these products and ease of their applications against their potential benefits to improve production performance and to increase overall profits. If growth performance, feed efficiency and mortality parameters are improved in commercial operations, then the costs of production are likely to be reduced. Moreover, if the chicken flock is better able to resist disease and survive until they are of marketable size, the subsequent cost of medication and overall production costs would be reduced considerably. As far as we know, this is the first study that evaluates the water administration of a prebiotic and its evaluation on performance and economic impact under commercial conditions.

In summary, the results of present study strongly suggest that this novel water applied prebiotic positively influences gastrointestinal integrity and positively influences in wide range of commercial parameters. Some of the limitations of the present pilot study are the number of samples and number of biomarkers used to assess gut barrier failure. Furthermore, microbiomics and metabolomics evaluation are required to generate a more comprehensive detailed understanding of the microbiota and metabolome in the gastrointestinal tract of chickens treated with this novel prebiotic. Studies to confirm and extend the evaluation of more biomarkers and the performance benefits in broiler chickens without antibiotic growth promoters in the diet are on-going.

## Data Availability Statement

The original contributions presented in the study are included in the article/supplementary material, further inquiries can be directed to the corresponding author/s.

## Ethics Statement

All animal studies followed guidelines as outlined by the Institutional Animal Care and Use Committee (IACUC) of the National Institute of Agronomic Technologies (INTA). The approved study protocol number is 03/2018.

## Author Contributions

IP-C and SL: conceptualization, methodology, software, supervision, and writing—original draft preparation. BV and EG: visualization, investigation, and data curation. BV, SL, JWH, and XH-V: final draft preparation, reviewing, and editing. All the authors reviewed and approved the manuscript.

## Conflict of Interest

IP-C, BV, and EG are employed by the Vetanco SA. SL is employed by Vetanco USA and BV Science. JWH is employed by Vetanco USA. The remaining author declare that the research was conducted in the absence of any commercial or financial relationships that could be construed as a potential conflict of interest.

## Abbreviations

BWG: body weight gain
FBW: final body weight
FCR: feed conversion ratio
FI: feed intake
GALT: gut-associated lymphoid tissue
IgA: immunoglobulin A
IL-8: interleukin 8
LAB: lactic acid bacteria
MOS: mannanoligosaccharides
MUC2: >mucin 2
sIgA: secretory immunoglobulin A
TGF-β4: transforming growth factor β4
TJ: tight junctions
ZO-1: zonula occludens-1.
